# Tumor Microenvironmental Competitive Endogenous RNA Network and Immune Cells Act as Robust Prognostic Predictor of Acute Myeloid Leukemia

**DOI:** 10.3389/fonc.2021.584884

**Published:** 2021-04-09

**Authors:** Yaqi Cheng, Xiaoran Wang, Peiyan Qi, Chengxiu Liu, Shoubi Wang, Qi Wan, Yurun Liu, Yaru Su, Lin Jin, Ying Liu, Chaoyang Li, Xuan Sang, Liu Yang, Chang Liu, Hucheng Duan, Zhichong Wang

**Affiliations:** ^1^ State Key Laboratory of Ophthalmology, Zhongshan Ophthalmic Center, Sun Yat-sen University, Guangzhou, China; ^2^ Guangzhou International Travel Health Care Center, Guangzhou, China; ^3^ Department of Ophthalmology, Affiliated Hospital of Qingdao University Medical College, Qingdao, China; ^4^ Department of Ophthalmology, The Second People’s Hospital of Foshan, Foshan, China

**Keywords:** acute myeloid leukemia, tumor microenvironment, ceRNA network, prognosis, immune microenvironment

## Abstract

Acute myeloid leukemia (AML) is malignant hematologic tumors with frequent recurrence and cause high mortality. Its fate is determined by abnormal intracellular competitive endogenous RNA (ceRNA) network and extracellular tumor microenvironment (TME). This study aims to build a ceRNA network related to AML TME to explore new prognostic and therapeutic targets. The RNA expression data of AML were obtained from The Cancer Genome Atlas (TCGA) database. First, we used the ESTIMATE algorithm to calculate the immune cells and stromal cells infiltration scores in the TME and found that all scores were highly correlated with AML’s prognostic characteristics. Subsequently, differentially expressed mRNAs and lncRNAs between high and low score groups were identified to construct a TME-related ceRNA network. Further, the Cox-lasso survival model was employed to screen out the hub prognostic ceRNA network composed of two mRNAs (EPB41L3, COL2A1), three miRNAs (hsa-mir-26a-5p, hsa-mir-148b-3p, hsa-mir-148a-3p), and two lncRNAs (CYP1B1-AS1, C9orf106), and construct nomograms. Finally, we used CIBERSORT algorithm and Kaplan-Meier survival analysis to identify the prognostic TME immune cells and found that naive B cells, M2-type macrophages, and helper follicular T cells were related to prognosis, and the hub ceRNAs were highly correlated with immune cell infiltration. This study provided a new perspective to elucidate how TME regulates AML process and put forward the new therapy strategies combining targeting tumor cells with disintegrating TME.

## Introduction

Acute myeloid leukemia (AML) is one of the most malignant hematologic tumors, characterized by the massive expansion of abnormally differentiated hematopoietic precursor cells (1, 2). AML shows a high degree of heterogeneity due to its complex genetic mutations and variable molecular phenotypes, which results in the failure of traditional treatment with a single mode of action in achieving an ideal effect (3). Although 50% of the AML patients could obtain a complete remission (CR) after receiving therapies, such as intensive induction chemotherapy and post-remission, still more than 20% of AML cases remain unresponsive and refractory. Even among patients who achieve CR, there is still a recurrence rate of up to 50% (4). Hematopoietic stem cell transplantation, the only method to cure AML currently, still faces severe challenges, such as tumor immune escape, recurrence, and graft-versus-host response (5). Targeted chemotherapy and chimeric antigen receptor cell therapy have brought a new breakthrough to the treatment of AML. But off-target, drug resistance, and even treatment-related death, still pose insurmountable obstacles to its safe application (6). Old age, high white blood cell counts, abnormal genotypes of leukemia cells, and complicating other myeloid diseases have significant effect on the prognosis of AML. To promote the transformation of prognostic markers, exploration into clinical application is the driving force for the leap-forward development of all tumor, including AML treatment strategies.

The generation and progression of tumor are determined by abnormal molecular aberrations inside tumor cells and the tumor microenvironment (TME) outside the cells. AML originates from abnormal bone marrow (BM) microenvironment. Tumor cells hijack and reshape the BM microenvironment ecology to transform it into tumor protective phenotype, mediating the immune escape and therapeutic tolerance of AML (7). Under the protection of abnormal BM microenvironment, AML stem cells and initiation cells can maintain their potential of regeneration, and small residual lesions can obtain effective incubation and induce the recurrence of AML (8). As a hotbed of AML, the abnormal BM microenvironment employs extracellular matrix (ECM) as a scaffold and contains cellular components, such as immune cells, stromal cells, endothelial cells, and various soluble molecules, such as exosomes, cytokines, and hormones. Among them, stromal cells and immune cells are key components that affect the progression of AML and therapeutic response.

The emergence of competitive endogenous RNA (ceRNA) network theory provides new ideas for exploring the internal mechanisms of tumorigenesis and development. Long non-coding RNA (lncRNA), circular RNA (circRNA), and other non-coding RNA (ncRNA) form an endogenous RNA competitive regulatory network when competing with mRNA for the binding to microRNA (miRNA). The competitive effect of ncRNAs is crucial to the structural stability and translation function of mRNAs. Abnormal ceRNA networks are involved in various tumor processes, including AML (9, 10). A number of studies have clarified the role of lncRNA and other ncRNAs or TME in AML and revealed their potential value in predicting the prognosis of AML (11, 12). Conducting in-depth exploration of the internal and external mechanisms of AML progress, looking for highly efficient prognostic targets, and combining precise targeting tumor cells with completely disintegrating tumor protective microenvironment may bring new breakthroughs for the treatment of AML.

In this study, we obtain the clinical information and transcriptome expression data of AML patients from The Cancer Genome Atlas (TCGA) database. The Estimate of STromal and Immune cells in MAlignant Tumor tissues using Expression data (ESTIMATE) was employed to quantify the scores of immune and stromal cells infiltrations in TME. DEmRNAs and DElncRNAs between high and low scores groups were identified, and the TME-related ceRNA network was constructed. We screened hub prognostic genes and established nomograms to quantify their predictive power. Meanwhile, The Cell Type Identification by Estimating Relative Subsets of RNA Transcripts (CIBERSORT) algorithm was used to calculate the composition and proportion of immune cells in TME, and the correlation between ceRNAs and immune cell infiltration was verified, so as to provide more reliable biological targets for AML therapy. Finally, we identified two lncRNAs (CYP1B1-AS1, C9orf106), three miRNAs (hsa-mir-26a-5p, hsa-mir-148b-3p, hsa-mir-148a-3p), and two mRNAs (EPB41L3 and COL2A1) to construct a prognostic lncRNA-miRNA-mRNA ceRNA network. Furthermore, our study identified that several immune cells, such as M2 macrophages, naive B cells, and helper follicular T cell, have dramatic prognosis value.

## Materials And Methods

### AML Transcriptome Data and Clinical Information

The RNA sequencing data and corresponding clinical information of 173 AML samples were obtained from the TCGA database (https://portal.gdc.cancer.gov/). The miRNA expression array GSE142699 (GPL26945 NanoString nCounter Human miRNA) downloaded from the Gene Expression Omnibus (GEO) database contains 24 AML and 24 normal control samples, and mRNA expression array GSE71014 (GPL10558 Illumina humanht-12 V4.0 expression beadchip) contains mRNA data and clinical information of 104 AML samples.

### Analysis of Differentially Expressed Genes Related to Microenvironment

The ESTIMATE algorithm can evaluate the non-tumor cell components in TME based on the gene expression characteristics of the tumor, and quantify the TME immune cell and stromal cell infiltration scores (13). ESTIMATE algorithm was used to analyze the AML mRNA sequencing data and calculate the BM microenvironment scores. The AML samples were divided into high, low immune infiltration groups and stromal infiltration groups with the median score as the boundary. The “limma” R package was used to identify differentially expressed mRNAs (DEmRNAs) and lncRNAs (DElncRNAs) between high- and low-stromal or immune score groups with |log2FC| > 1 and adjusted P value < 0.05 as the cutoff criteria, and then intersection was taken (14, 15). Meanwhile, differentially expressed miRNAs (DEmiRNAs) between AML and normal samples were identified after normalization, |log2FC|>1 and adjusted P value <0.05 was defined as the cutoff criteria (16, 17). The “pheatmap” and “ggplot2” R packages were involved in drawing heatmap and volcanoes of the differential genes.

### Gene Ontology and Kyoto Encyclopedia of Genes and Genomes Analysis

The “clusterProfiler” (18) R package was used to perform gene ontology (GO) enrichment analysis of DEmRNAs to reveal the biological processes (BP), cellular components (CC) and molecular functions (MF) that they are involved in. Kyoto encyclopedia of genes and genomes (KEGG) enrichment analysis was used to annotate the signaling pathways DEmRNAs involved. The adjusted P value <0.05 was statistically significant.

### CeRNA Network Construction

MiRcode (19) database was employed to predict the miRNAs (lnc-pre-miRNAs) targeted by DElncRNAs. The intersection of lnc-pre-miRNAs and DEmiRNAs was obtained, and then the mRNAs (mi-pre-mRNAs) targeted by the intersecting miRNAs were predicted through Targetscan (20)and miRDB (21) databases. The intersection was taken within mi-pre-mRNAs and DEmRNAs. miRNAs and lncRNAs related to common mRNAs were identified according to the targeting relationship, and Cytoscape v3.7.2 was used to construct the initial TME-related ceRNA network.

### Cox, Lasso Regression Analysis, and Construction of Nomogram

The Cox-lasso survival model was constructed to screen the hub prognostic mRNAs. Univariate Cox proportional hazards regression analysis was employed to identify the relationship between the mRNAs expression and overall survival (OS) of patients using the “survival” R package, and the forest map was drawn using the “forestplot” R package. The mRNAs with P<0.05 entered lasso regression and multivariate Cox proportional hazards regression, which were also performed with the “survival” R package. Subsequently, with the median of the risk scores obtained by multivariate Cox regression as the boundary, the AML patients were divided into high and low risk groups. The “survival” R package was employed to perform survival analysis and draw survival curves of the two groups. At the same time, the hub prognostic mRNAs nomogram was established based on the results of multivariate Cox regression to quantitatively predict the prognosis of AML, using the “rms” R package.

### TME Immune Cell Infiltration Analysis

CIBERSORT algorithm analyzes gene expression data to identify cell abundance and proportion in mixed cell populations. CIBERSORT can easily recognize each cell type and its count in each sample (22). We used CIBERSORT algorithm to identify the proportion of the 22 types of immune cells infiltrated in AML samples. Analyses were performed with 100 permutations with the default statistical parameters to improve the credibility of the results. Samples with a CIBERSORT output of P < 0.05 were considered to be eligible for further analysis (16, 23).

### Statistical Analysis

The Kaplan‐Meier survival analysis was performed using the R package “Survival” to analyze the correlations between patients’ OS and other variates, such as TME scores, hub prognostic mRNAs’ expression, and immune cell infiltration proportion, and also employed in identifying the OS’ difference between high and low risk groups after Cox regression, two-sided P <0.05 was the statistically significant cutoff. The statistical significance of the correlation was tested by the log-rank test. Pearson correlation analysis was done for each prognostic biomarker in the ceRNA network and the proportion of each microenvironment related immune cell using the “limma” package (23). All statistical analyses were performed by R software (v.3.6.3) and corresponding program packages.

## Results

### TME Scores Were Highly Related to the AML Patients Prognosis

**Figure 1:**
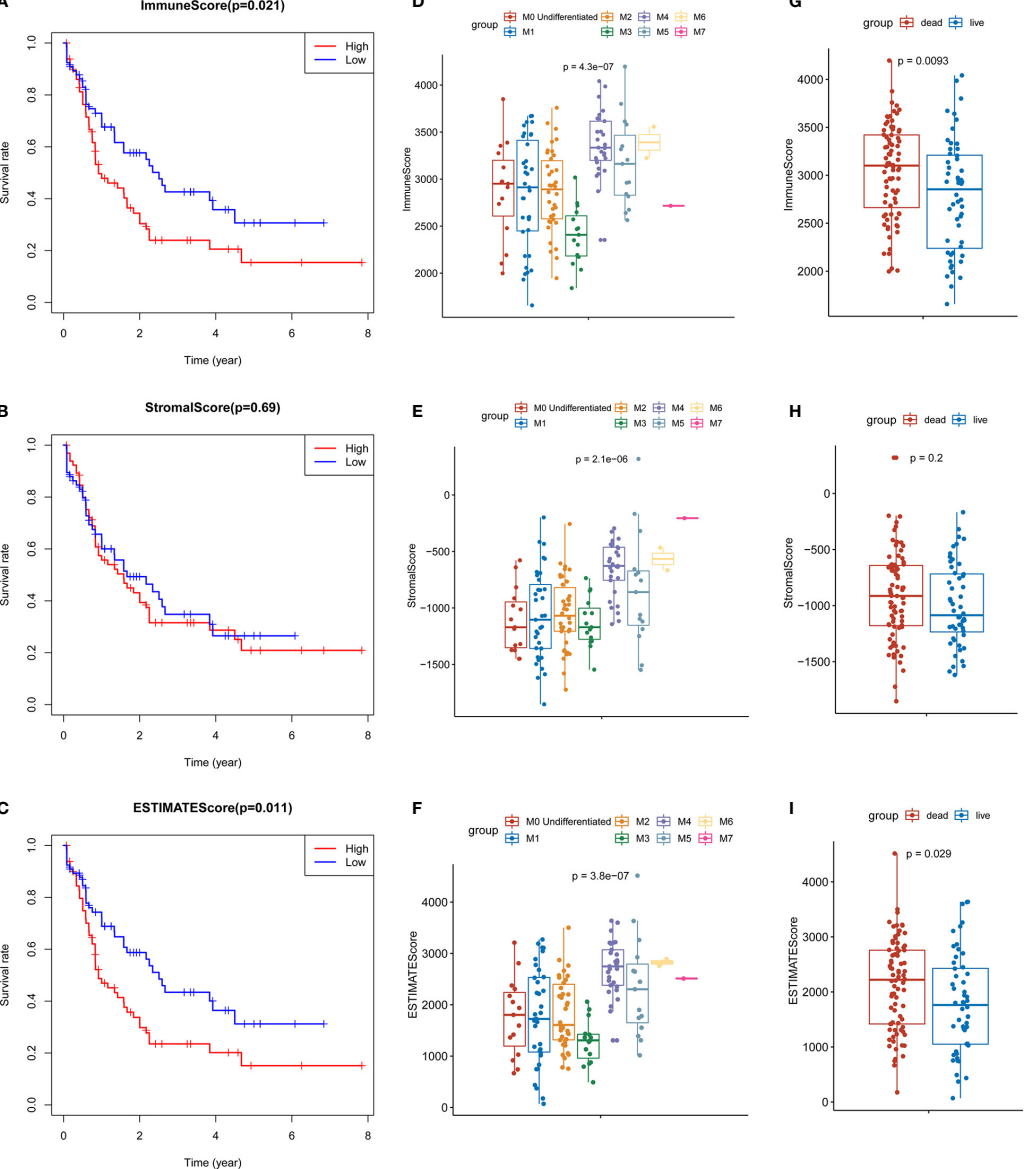
Identification of the relationship between TME scores and prognosis of AML. **(A)** Survival curve of high and low immune groups: the survival rate of patients with high immune scores was significantly reduced (P = 0.021). **(B)** Survival curves of high and low stromal groups: the survival rate of patients with high stromal score decreased, with no significant statistical difference (P = 0.69). **(C)** Survival analysis showed that the survival rate of patients with high ESTIMATE score was significantly reduced (P = 0.011). **(D–F)** The distribution of the three scores in different FAB classifications are significantly different (all P values < 0.0001); **(G–H)** The relationship between the survival status of AML patients and three scores: the immune scores of the dead cases (P = 0.0093) and the ESTIMATE scores (P = 0.029) were significantly higher than those of the surviving patients, and the patients with high stromal scores were more likely to be in the state of death, but there was no significant statistical difference (P = 0.2).

The results of ESTIMATE analysis showed that the immune scores of 173 AML samples ranged from 1329.53 to 3971.97, and the stromal scores ranged from −1888.81 to 435.75. The ESTIMATE scores were the sum of immune scores and stromal scores. The AML patients were divided into high and low groups according to stromal scores, immune scores, and ESTIMATE scores to perform survival analysis. Results showed that the survival time of the patients with high immune scores (P = 0.021; **Figure 1A**) and high ESTIMATE scores (P = 0.011; **Figure 1C**) is much shorter than those with the low scores. Meanwhile, the overall survival time of patients with high stromal scores was shortened, but there was no significant statistical difference (P = 0.69; **Figure 1B**).

At the same time, we analyzed the relationship between three types of scores and prognosis-related clinical traits. The results showed that immune, stromal, and ESTIMATE scores were all significantly different among different FAB classification of leukemia (P < 0.001; **Figures 1D–F);** The three scores were also correlated with the survival status of the patients. Each score in the death cases was significantly increased, among which stromal score were not statistically significant (**Figures 1G–I**). In addition, immune scores were significantly different in different cytogenetic risk classifications (P = 0.011; **Supplementary Figures 1A–C**), while the three scores were not related to gender (P >0.05; **Supplementary Figures 1D–G**). All the above results indicate that TME immune cells and stromal cells are of great significance in the prognosis of AML, especially in the diagnosis and classification of AML.

### Identification of TME-Related DEmRNAs and DElncRNAs

**Figure 2:**
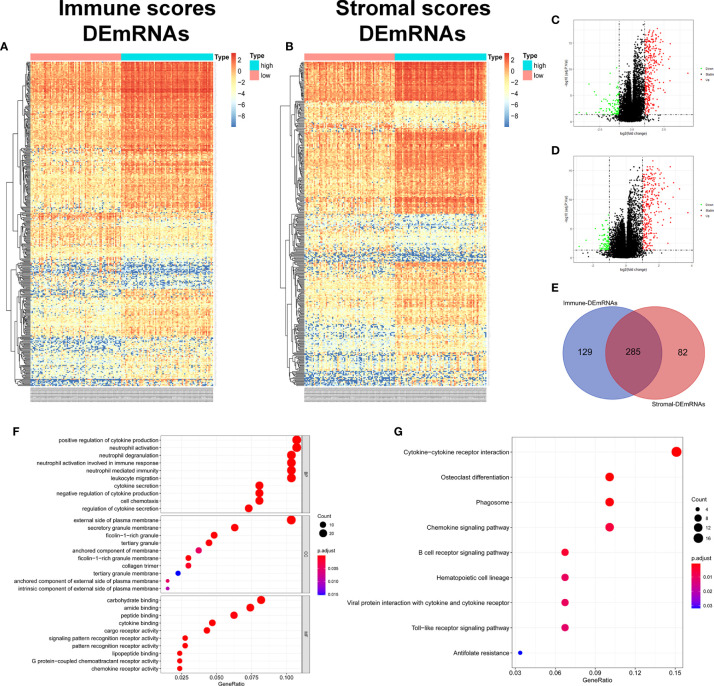
Identification of TME-related mRNAs. **(A)** Heatmap of DEmRNAs between high and low immune groups. **(B)** Heatmap of DEmRNAs between high and low stromal groups. **(C)** Volcano map of mRNAs expression between high and low immune groups. **(D)** Volcano map of mRNAs expression between high and low stromal groups. **(E)** Venn diagram was used for the intersection of DEmRNAs within immune and stromal groups, we considered the common DEmRNAs to be TME-related mRNAs. **(F)** GO enrichment analysis was performed to annotate TME-related DEmRNAs: the larger the bubble and longer columns represent the more genes enriched in this function, the deeper the color of the bubble and bars, the smaller the P value. **(G)** TME-related DEmRNAs KEGG enrichment analysis results.

**Figure 3:**
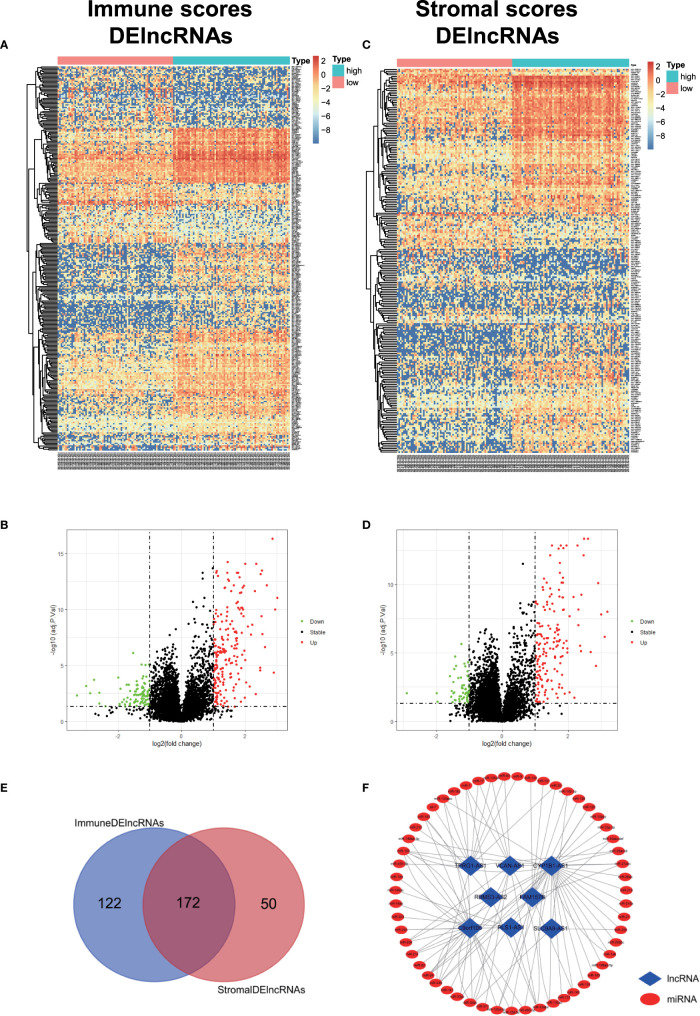
Identification of TME-related lncRNAs. **(A–D)** Heatmaps and volcano maps of DElncRNAs between high and low immune and stromal groups. **(E)** Venn diagram was used for the intersection of DElncRNAs within immune and stromal groups, and we considered the common DElncRNAs to be TME-related mRNAs. **(F)** miRNAs predicted by TME-related lncRNAs.

In order to evaluate the possible impact of stromal and immune scores on breast cancer, we investigated the expression patterns in different stromal and immune groups. DEmRNAs and DElncRNAs were compared between high- and low-stromal or immune score groups with |log2FC| > 1 and adjusted P value < 0.05 as the cutoff criteria through R package “limma.” For comparison based on immune scores, there were 414 DEmRNAs (**Figures 2A, C**) and 294 DElncRNAs (**Figures 3A**, **B**) in the high immune-score group. Similarly, for the high and low groups based on stromal scores, 367 DEmRNAs (**Figures 2B, D**) and 222 DElncRNAs (**Figures 3C, D**) were obtained. After taking the intersection of mRNAs and lncRNAs separately, we got 285 mRNAs (**Figure 2E**) and 172 lncRNAs (**Figure 3E**) that were differentially expressed in in both stromal and immune groups. We believed that these genes were highly correlated with TME and used them for further analysis.

285 common DEmRNAs were subjected to perform GO and KEGG enrichment analysis to further verify the biological processes involved. The results of GO analysis showed that they were mainly enriched in cytokine production and secretion (GO: 0050663, GO: 0001819, GO: 0050707), neutrophil activation (GO: 0042119, GO: 0043312), lymphocytes (GO: 0050900), and T Cell (GO: 0042110) function and immune response (GO: 0050727, GO: 0045088) and other immune processes (**Figure 2F**, **Table 1**). KEGG analysis showed they were enriched in signaling pathways, such as osteoclast differentiation (hsa04380), cytokines and cytokine receptor interaction (hsa04060), phagosome (hsa04145), and B cell receptor (hsa04662) (**Figure 2G**, **Table 2**). Both are related to tumor immune response and TME remodeling.

**Table 1:** GO enrichment analysis results of the TME-related DEmRNAs.

Ontology	Term	Description	Gene Count	adj. P value
BP	GO:0050663	cytokine secretion	21	9.99E-08
BP	GO:0001819	positive regulation of cytokine production	28	1.69E-07
BP	GO:0050707	regulation of cytokine secretion	19	1.69E-07
BP	GO:0042119	neutrophil activation	28	4.34E-07
BP	GO:0043312	neutrophil degranulation	27	7.32E-07
CC	GO:0009897	external side of plasma membrane	28	4.09E-10
CC	GO:0030667	secretory granule membrane	17	0.00014
CC	GO:0101003	ficolin-1-rich granule membrane	8	0.00014
CC	GO:0101002	ficolin-1-rich granule	13	0.00014
CC	GO:0070820	tertiary granule	12	0.00018
MF	GO:0030246	carbohydrate binding	21	2.78E-07
MF	GO:0071723	lipopeptide binding	6	4.63E-07
MF	GO:0008329	signaling pattern recognition receptor activity	7	1.45E-06
MF	GO:0038187	pattern recognition receptor activity	7	1.61E-06
MF	GO:0038024	cargo receptor activity	11	4.37E-06

**Table 2:** KEGG enrichment analysis results of the TME-related DEmRNAs.

TermID	Description	Gene Count	adj.P value
hsa04380	Osteoclast differentiation	12	0.00065
hsa04060	Cytokine-cytokine receptor interaction	18	0.00071
hsa04145	Phagosome	12	0.00124
hsa04662	B cell receptor signaling pathway	8	0.00453
hsa04062	Chemokine signaling pathway	12	0.00603
hsa04640	Hematopoietic cell lineage	8	0.01007
hsa04061	Viral protein interaction with cytokine and cytokine receptor	8	0.01007
hsa04620	Toll-like receptor signaling pathway	8	0.01143
hsa01523	Antifolate resistance	4	0.03182

### Identification of DEmiRNAs and Preliminary Construction of TME-Related ceRNA Network

**Figure 4:**
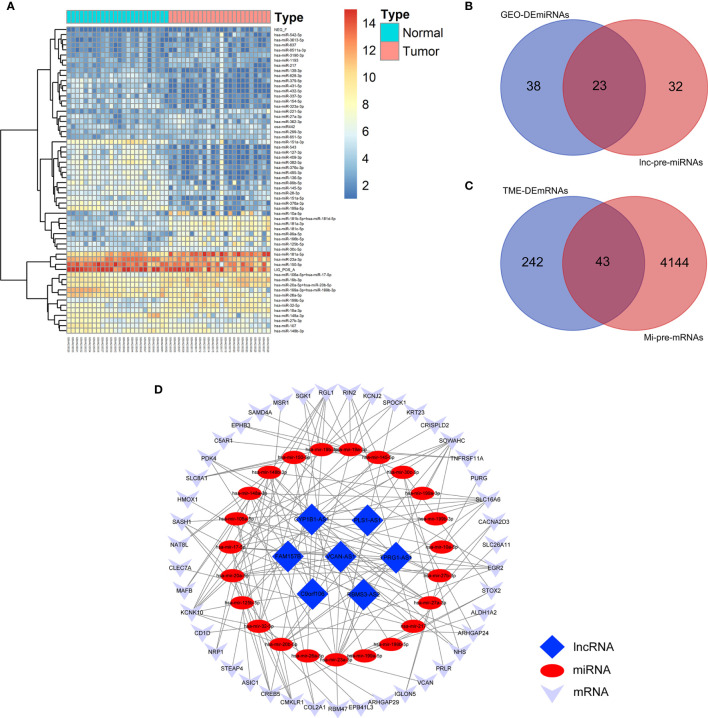
Construction of TME-related ceRNA network. **(A)** Heatmap of DEmiRNAs between AML and normal control samples in GSE142699 microarray. **(B)** Venn diagram of the intersecting miRNAs between DEmiRNAs and lnc-pre-miRNAs. **(C)** Venn diagram of the intersecting of mRNAs between mi-pe-mRNAs and TME-related DEmRNAs, a total of 43 common mRNAs. **(D)** Preliminary establishment of TME-related ceRNA network, including 43 mRNAs, 23 miRNAs, and 7 lncRNAs.

We obtained the DEmiRNAs between the 24 AML and 24 normal control samples in GSE142699 miRNA microarray. A total of 61 DEmiRNAs were identified with |log2FC|>1 and adjusted P value <0.05 as the cutoff criteria (**Figure 4A**).

We predicted the targeting relationships among lncRNAs, miRNAs and mRNAs through a variety of databases and preliminarily established TME-related ceRNA network.

First, 55 lnc-pre-miRNAs were obtained by predicting the 172 common DelncRNAs targeting genes (**Figure 3F**). The intersection of lnc-pre-miRNAs and DEmiRNAs yielded 23 miRNAs (**Figure 4B**). Subsequently, 4187 mi-pre-mRNAs targeted by the 23 intersecting miRNAs were predicted by both Targetscan and miRDB databases (**Figure 4C**). In order to improve the correlation between ceRNA network and TME, the intersection of mi-pre-mRNAs and DEmRNAs was taken, and 43 hub mRNAs were obtained. Finally, we preliminarily constructed a TME-related ceRNA network consisting of 43 mRNAs, 23 miRNAs and 7 lncRNAs (**Figure 4D**). GO and KEGG enrichment analysis were performed on 43 mRNAs to verify their involvement in TME and immune-related processes (**Supplementary Figure 2**).

### Survival Model to Screen Hub Prognostic mRNAs and Construct Nomograms

**Figure 5:**
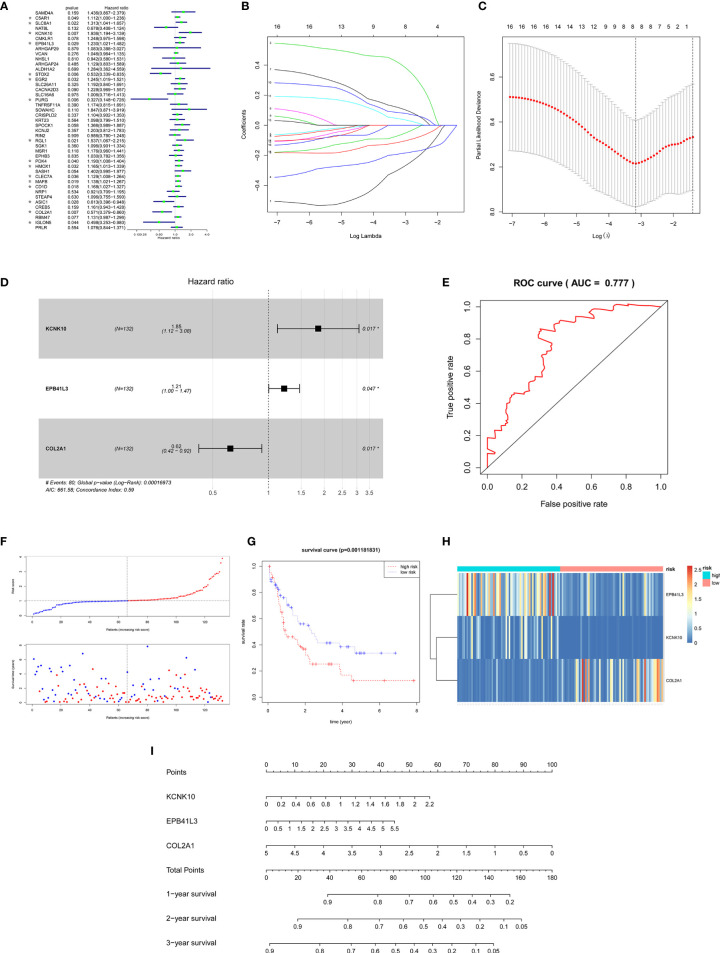
Construction of the survival model to screen hub prognostic mRNAs. **(A)** Forest map of univariate Cox proportional hazards regression: 16 mRNAs with *P* < 0.05 were screened out of 43 common mRNAs (marked by *). **(B, C)** Lasso regression screening of mRNAs into multivariate Cox regression: λ and cv graphs show that eight hub mRNAs were selected. **(D)** Forest map of multivariate Cox proportional hazards regression: three hub prognostic mRNAs (KCNK10, EPB41L3, COL2A1) were screened out. **(E)** ROC curve of multivariate Cox regression: AUC of 5-year survival was 0.777, indicating the high accuracy of multivariate Cox regression model. **(F)** The AML samples were divided into high and low risk groups according to the risk scores obtained from Cox regression. **(G)** Survival curve of high and low risk groups: the survival rate of the high-risk group was significantly reduced (P = 0.0011). **(H)** Heatmap of KCNK10, EPB41L3, and COL2A1 expressions among the high and low risk group samples. **(I)** Nomogram of KCNK10, EPB41L3, and COL2A1 based on the results of the multivariate Cox regression.

In order to ensure the accuracy and sensitivity of AML prognosis prediction, we constructed a survival model consisting of univariate Cox proportional hazard regression-lasso regression-multivariate Cox proportional hazard regression to further screen the hub prognostic ceRNAs. Firstly, 16 of 43 common mRNAs which were highly related to overall survival (OS) of AML patients were screened by univariate Cox regression (P <0.05; **Figure 5A**). To prevent overfitting of multivariate Cox regression, Lasso regression was used to screen eight mRNAs from 16 mRNAs (**Figures 5B, C** **)**. Three hub prognostic mRNAs: KCNK10, EPB41L3, and COL2A1 were finally screened out by multivariate Cox regression (**Figure 5D**, **Table 3**). The receiver operating characteristic (ROC) curve was drawn to check the accuracy of the model. The area under the curve (AUC) of 5-year survival was 0.777, indicating the high accuracy of this model (**Figure 5E**). Kaplan-Meier survival analysis of high and low risk groups showed that the survival rate of the high-risk group was significantly lower than that of the low-risk group (P = 0.001; **Figures 5F, G** **)**. Simultaneously, heatmap of the differential expression of three hub mRNAs between the high and low risk groups were drawn (**Figure 5H**), and a nomogram was constructed according to the expression of the three mRNAs (**Figure 5I**).

**Table 3:** Identification of hub prognostic mRNAs through univariate Cox regression-lasso regression-multivariate Cox regression.

Gene	Univariate Cox		LASSO		Multivariate Cox	
	HR	P value	Coef	HR	P value	Coef
PURG	0.32741	0.006	−0.28865			
STOX2	0.53225	0.00603	−0.1104			
KCNK10	1.93618	0.00737	0.40878	1.85342	0.0172	0.61703
COL2A1	0.57073	0.0074	−0.18884	0.62192	0.01738	−0.47494
CD1D	1.16757	0.01775				
MAFB	1.13763	0.01911				
RGL1	1.53696	0.02108	0.11465			
SLC8A1	1.31335	0.0217				
ASIC1	0.6127	0.02769				
EPB41L3	1.23016	0.02918	0.08386	1.21387	0.04665	0.19382
EGR2	1.24481	0.03204	0.0429			
HMOX1	1.16491	0.03215				
CLEC7A	1.12871	0.03597				
PDK4	1.18969	0.04026				
IGLON5	0.49832	0.0436	−0.06407			
C5AR1	1.11199	0.04958				

**Figure 6:**
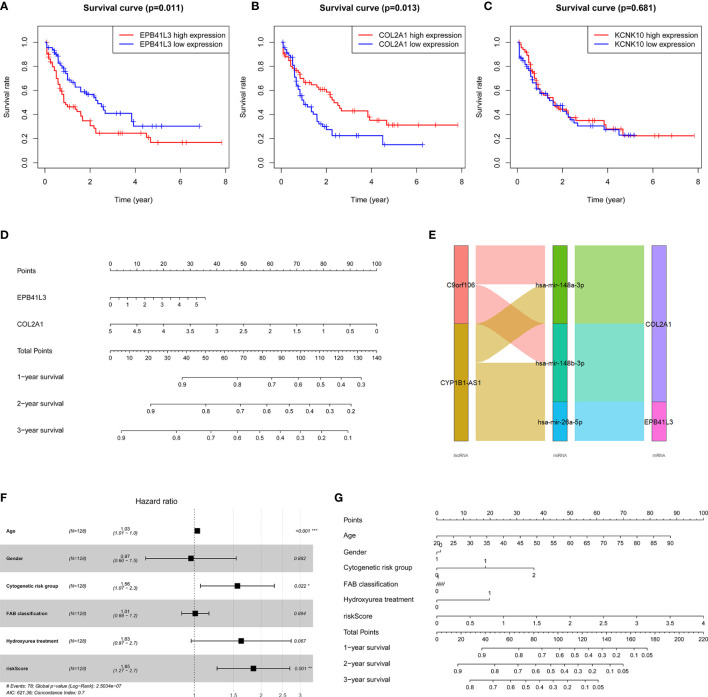
Verifying the prognostic value of the three core mRNAs. **(A–C)** Survival curve of the three hub mRNAs: the survival rate of AML patients with high expression of EPB41L3 significantly decreased (P = 0.011), and the survival rate of AML patients with low expression of COL2A1 significantly decreased (P = 0.013). There is no significant difference in survival rate between different KCNK10 expression levels (P = 0.681), so we believe that EPB41L3 and COL2A1 are core prognostic mRNAs with predictive value. **(D)** Nomogram of EPB41L3 and COL2A1 based on the results of the multivariate Cox regression. **(E)** Hub prognostic ceRNA network based on EPB41L3 and COL2A1; **(F)** Forest map of multivariate Cox regression analysis: the risk score of prognostic model maintained independence in predicting the OS of AML patients (HR = 1.8469, 95% CI = 1.2652–2.6961, P = 0.00147). At the same time, age and cytogenetic risk groups of AML patients also have independent prognosis value. **(G)** The nomogram of the risk score of prognostic model and clinic characters.

At the same time, we performed Kaplan-Meier survival analysis of the three hub mRNAs separately. The results showed that the survival rate of patients with high EPB41L3 expression was significantly reduced (P = 0.011; **Figure 6A**), and low expression of COL2A1 predicted poor prognosis (P = 0.013; **Figure 6B**), while there was no significant relationship between the KCNK10 expression and the prognosis of AML (P = 0.681; **Figure 6C**). The survival analysis results of EPB41L3, COL2A1, and KCNK10 in GSE71014 array were consistent with the above, but there was no significant statistical difference in the survival analysis of the three mRNAs (**Supplementary Figure 3**). Therefore, we identified EPB41L3 and COL2A1 as the hub prognostic mRNAs and plotted the prognostic nomogram of the two genes (**Figure 6D**). Finally, the hub ceRNA network consisting of two mRNAs (EPB41L3 and COL2A1), three miRNAs (hsa-mir-26a-5p, hsa-mir-148b-3p, hsa-mir-148a-3p), and two lncRNAs (CYP1B1-AS1, C9orf106) were constructed (**Figure 6E**).

Furthermore, in order to evaluate the independent predictive value of the two hub mRNAs prognostic model in patients with complete clinical information from the TCGA cohort, we constructed univariate and multivariate Cox regression analyses according to the risk scores from prognostic model and clinical covariates including age, gender, cytogenetic risk group, FAB classification, history of hydroxyurea treatment. The result of univariate Cox regression analysis revealed that risk scores have obvious predictive value (*P* < 0.0001), and some clinic characters, such as age (*P* < 0.0001) and cytogenetic risk group (*P* < 0.001) also had prognosis significance (**Table 4**). The multivariate Cox regression analysis indicated that the risk score of prognostic model maintained independence in predicting the OS of AML patients (HR = 1.8469, 95% CI = 1.2652–2.6961, P = 0.00147) (**Figure 6F**, **Table 4**). We plotted the prognostic nomogram of the risk score of prognostic model and clinic characters (**Figure 6G**).

**Table 4:** Identification the independent prognostic value of survival model and clinic characters through univariate Cox regression and multivariate Cox regression.

Marker	Univariate Cox				Multivariate Cox			
	HR	HR.95L	HR.95H	P value	HR	HR.95L	HR.95H	P value
Age	1.0348	1.0188	1.0512	<0.0001	1.0312	1.0147	1.0479	0.0002
Gender	0.9956	0.6364	1.5578	0.9847	0.9651	0.6028	1.5452	0.8824
Cytogenetic Risk Group	1.7833	1.2663	2.5116	0.0009	1.5639	1.0662	2.2939	0.0221
FAB Classification	1.0761	0.9324	1.2418	0.3159	1.0097	0.8762	1.1636	0.8937
Hydroxyurea Treatment	1.6259	0.9849	2.6842	0.0573	1.6257	0.9671	2.7326	0.0667
Risk Score	2.0212	1.4738	2.7722	<0.0001	1.8469	1.2652	2.6961	0.0015

### AML Microenvironment Immune Cell Infiltration and Its Prognostic Value

**Figure 7:**
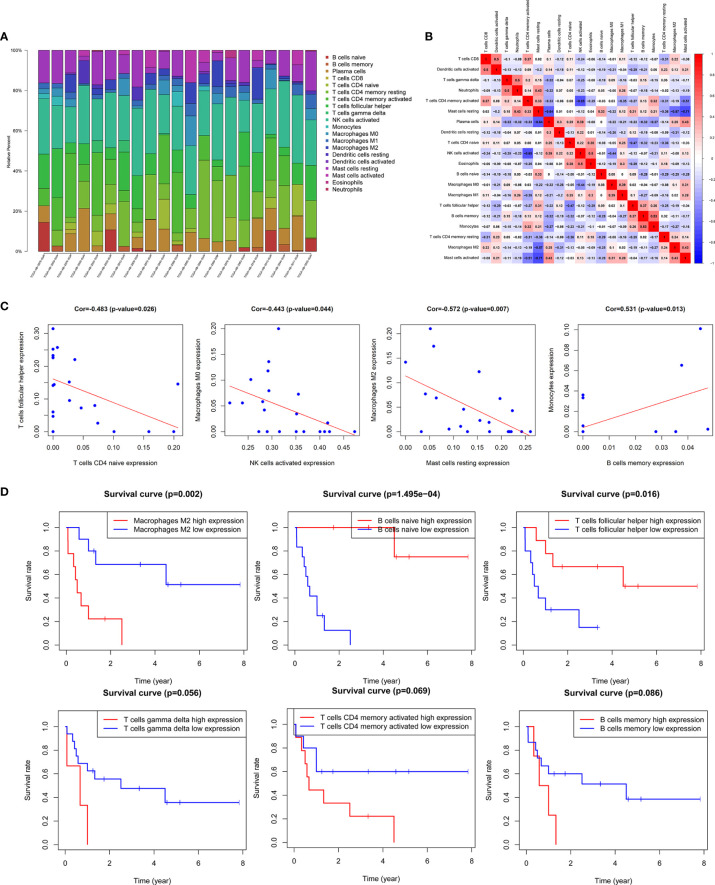
Correlation analysis of TME immune cells and prognosis. **(A)** Histogram of the infiltration proportion of 22 types of immune cells between different samples. **(B)** Correlation heat map between immune cells. **(C)** Plots of correlation between immune cells: memory B cells and monocytes (P = 0.013) was significantly positively correlated; there was a significant negative correlation between resting mast cells and M2 macrophages (P = 0.007), activated NK cells and M0 macrophages (P = 0.044), naive CD4 memory T cells and helper follicular T cells (P = 0.026). **(D)** Survival curves of TME immune cell: the survival rate of AML patients with high proportion of M2 macrophages infiltration was significantly reduced (P = 0.002), patients with low proportion of naive B cells (P <0.001) and helper follicular T cells (P = 0.016) had decreased survival rate. In addition, the increase in the ratio of γδ T cells, activated CD4 memory T cells and memory B cells also predicted the adverse outcome of AML, with no statistically significant difference.

In this study, the CIBERSORT algorithm was used to analyze the 22 immune cell subtypes infiltration in the AML microenvironment (**Figure 7A**) and the infiltrating differences between the high and low immune and stromal score groups obtained by the ESTIMATE algorithm were analyzed (**Supplementary Figure 4**). The results showed that the resting dendritic cells, resting mast cells and neutrophils were significantly different between different immune and stromal groups (P <0.05), and the activated mast cells (P = 0.013) were significantly different between the high and low stromal groups (P = 0.013). Then Pearson test was performed to identify the correlation between TME immune cells and the correlation heatmap was drawn (**Figure 7B**). The results showed that there were strong correlations among various immune cells such as M2 macrophages, activated NK cells, memory B cells, and helper follicular T cells (**Figure 7C**, **Supplementary Figure 5**).

The results of Kaplan-Meier survival analysis showed that the M2 macrophages (P = 0.002), naive B cells (P <0.001), helper follicular T cell (P = 0.016) were significantly correlated with the survival rate of patients. In addition, the increased proportion of γδT cells, activated CD4 memory T cells and memory B cells also predicted a poor outcome, but the results were not statistically significant (**Figure 7D**, **Supplementary Figure 6**).

### Correlation Between TME-Related Hub Prognosis ceRNA Network and Immune Cell Infiltration

**Figure 8:**
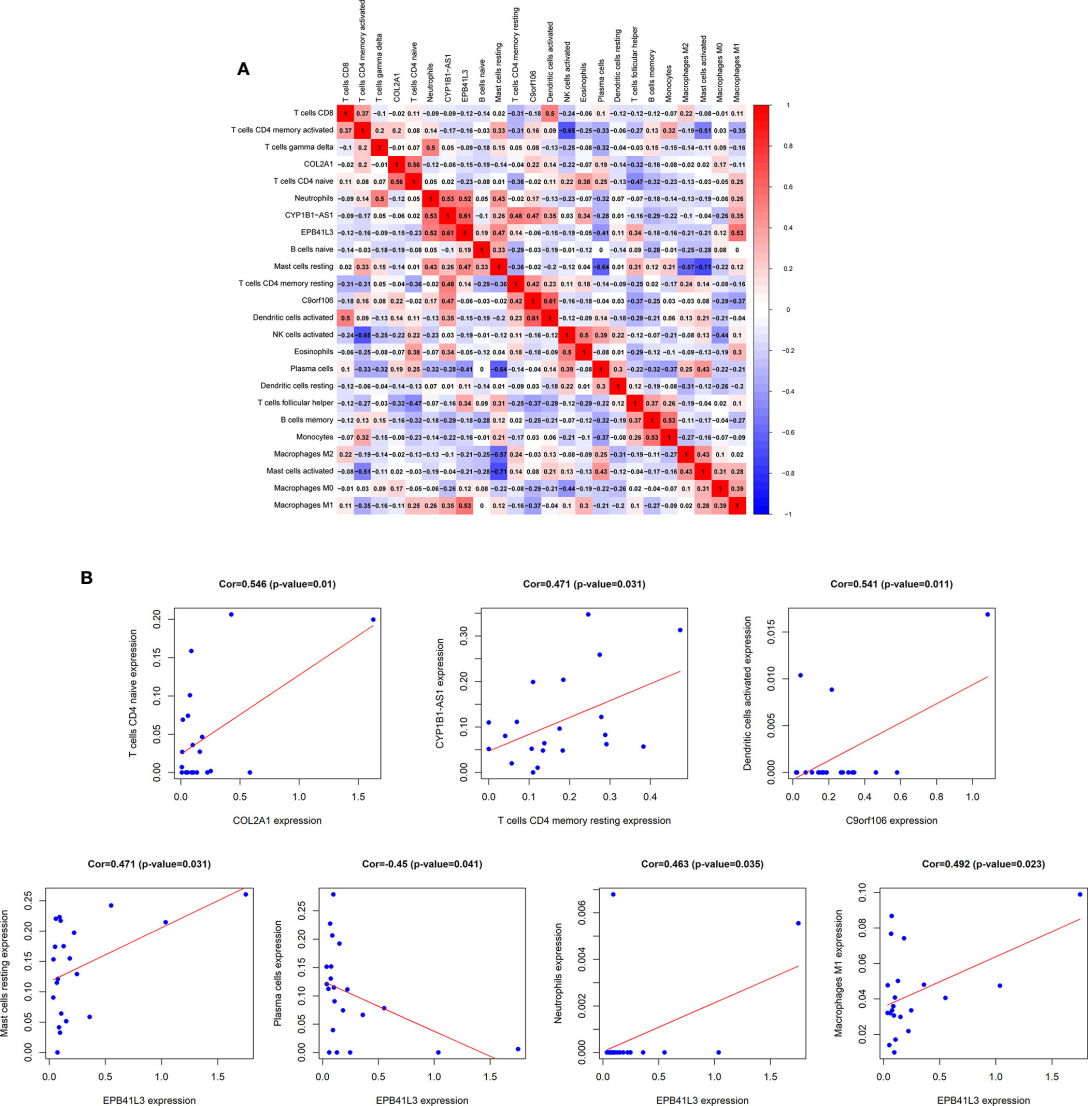
Correlation analysis of hub prognostic ceRNA network and TME immune cells. **(A)** Correlation heat map between EPB41L3, COL2A1, CYP1B1-AS1, C9orf106, and TME infiltrating immune cells; **(B)** Verifying the correlation between EPB41L3, COL2A1, CYP1B1-AS1, C9orf106, and immune cells: EPB41L3 was significantly positively correlated with resting mast cells, neutrophils, and M1 macrophages, and had strong negative correlation with plasma cells, COL2A1 was positively correlated with naive CD4 T cells. CYP1B1-AS1 was positively correlated with resting CD4 memory T cells. C9orf106 was positively correlated with activated dendritic cells (all P values <0.05).

In order to fully elucidate the mechanism of the hub prognostic ceRNA network in AML, and at the same time look for the potential way for TME to determine the fate of AML, we performed Pearson test on the correlation between EPB41L3, COL2A1, CYP1B1-AS1, C9orf106 and TME immune cells and plotted heatmap (**Figure 8A**). The results showed that EPB41L3, COL2A1, CYP1B1-AS1, and C9orf106 were all related to TME immune cells. EPB41L3 was significantly positively correlated with resting mast cells, neutrophils and M1 macrophages, while being negatively correlated with plasma cells; COL2A1 was positively correlated with naive CD4 T cells; CYP1B1-AS1 was positively correlated with resting CD4 memory T cells, and C9orf106 was positively correlated with activated dendritic cells (all P values < 0.05; **Figure 8B**). In addition, we found that the expression of CYP1B1-AS1 was significantly positively correlated with that of the EPB41L3 and C9orf106 (**Supplementary Figure 7**).

## Discussion

AML progresses rapidly and causes a very high mortality rate. Its initiation, development, drug resistance and recurrence all depend on the abnormal molecular and genetic changes inside the cell and the protection of the extracellular TME (24). More and more scholars have realized that current immune therapies and targeted therapies cannot fully deal with the extremely complex heterogeneity of AML, and are committed to excavating new prognostic biomarkers to promote the innovation of AML precision diagnosis and treatment (25, 26). This study identified the TME related hub prognostic lncRNA-miRNA-mRNA ceRNA network, while focusing on the correlations between specific TME immune cell populations and ceRNA network of AML. A multiple prognostic verification model based on TME score survival analysis-ceRNA survival model-TME immune cell infiltration survival analysis was constructed, which highly improved the prognosis predicting accuracy of the biomarkers in this study while ensuring the high correlation between ceRNA and TME. We tried to elucidate the mechanism of TME regulating the fate of AML, and provide a unique perspective for finding new targets for AML.

AML cells manipulate TME through a complex interaction network, domesticate and reshape it into a pro-leukemia phenotype. The modified AML protective TME in turn promotes AML progression, providing tumor cells with an increasingly strong bastion, and forming a positive feedback loop of tumor-TME mutually promoting. Stromal cells and immune cells in TME have important regulatory and protective effects on AML. Abnormally expressed adhesion molecules, cell cycle regulators and angiogenic factors in tumor-associated stromal cells promotes the angiogenesis in TME, and meanwhile enable AML cells to accelerate proliferation, resist apoptosis and malignantly invade (27). TME immune cell-mediated inflammatory response is considered to be an important driving force for the remodeling of the AML microenvironment (28). In this study, ESTIMATE algorithm was employed to identify the infiltrating immune and stromal cells in the AML TME, and it was confirmed that the infiltration degree of these two types of cells was significantly correlated with the prognosis of AML, and it had a prominent effect on assisting the diagnosis of AML FAB typing. At the same time, the TME related mRNAs and lncRNAs were obtained according to the TME score grouping and the ceRNA network was constructed. These results and opinions are consistent with the findings of Yan (11), Huang (29) and others who utilized the ESTIMATE algorithm to identify hub prognostic mRNAs related to the AML microenvironment.

As an important post-transcriptional regulatory mechanism, ceRNA network is of great significance in the progression of AML. Several studies have proved that HOXA-AS2, RPPH1, and other lncRNAs regulate mRNAs expression as ceRNA and participate in AML proliferation, differentiation, and invasion (10, 30, 31). We constructed a survival prediction model of univariate Cox regression-lasso regression-multivariate Cox regression, supplemented by Kaplan-Meier survival analysis and nomograms, to identify hub mRNAs (EPB41L3, COL2A1) with both high TME correlation and high prognostic efficacy and construct the hub prognostic ceRNA network by layer-upon-layer screening. As early as 2003, Celal et al. reported the high expression of EPB41L3 in AML cell line HL-60 (32), which was consistent with the poor prognosis of AML patients with up-regulated EPB41L3 expression in this study. As the gene involved in cytoskeletal construction, EPB41L3 has also been shown to promote tumor metastasis by promoting epithelium-mesenchymal transformation in advanced lung cancer (33). The dynamic balance of osteoblasts and osteoclasts is of great significance for maintaining the normal BM microenvironment. Increased osteoclast activity in the BM of AML patients results in bone demineralization and the destruction of the normal BM structure (34). COL2A1 encodes type II collagen α1 chain, which participates in the composition of ECM, is an important osteogenic protein. Decreased expression of COL2A1 reduces the osteogenic protein, destroys normal ECM structure, promotes the remodeling of the BM microenvironment toward the direction of tumor promotion, and participates in the mutual promotion between TME and AML (35, 36). Ganapathi et al. demonstrated that the low expression of COL2A1 was significantly associated with the rapid recurrence of high-grade serous breast cancer, and proposed that the tumor suppressive effect of COL2A1 might be achieved by depleting oncogene miR-301 as ceRNA (37), revealing the important role of COL2A1 related ceRNA network in tumor development. Meanwhile, miRNAs in this study have been proved to be closely related to AML. The decreased expression of hsa-mir-26a-5p in AML can cause high expression of peroxiredoxin III, thereby promptly clearing the reactive oxygen species within cells and protecting tumor cells from oxidative stress injury (38, 39); In another study, researchers found that enforced expression of hsa-mir-26a-5p in AML cells was able to inhibit cell cycle progression by downregulating cyclin E2 expression, potentiated the antiproliferative effects of 1,25-dihydroxyvitamin D (3) (VitD) and stimulated myeloid differentiation by targeting E2F7 (40). Furthermore, hsa-mir-26a-5p was also proved as a target of c-Myc, which revealed the vital role this miRNA played in ceRNA network (41). Huang et al. demonstrated that MLL-fusion/MYC⊣miR-26a⊣TET1 signaling circuit played an important role in AML, in which hsa-mir-26a-5p functioned as an essential tumor-suppressor mediator and its transcriptional repression was required for the overexpression and oncogenic function of TET1 in MLL-rearranged AML (42). miRNAs sequencing of exosomes derived from bone marrow mesenchymal stromal cells and bone marrow specimens also found hsa-mir-26a-5p to be significantly associated with overall survival of AML patients and was closely related to HOX-related genes (43). Wang et al. found that the expression of hsa-mir-148 family decreased in AML patients, and it was highly correlated with FAB typing of AML (44).They also verified that DNMT1 was identified to be a downstream target of hsa-mir-148, and was negatively regulated by miR-148a in AML cell lines. It was a strong evidence that hsa-mir-148 was involved in the regulation of AML as a core member of ceRNA network (45). However, the role of two hub lncRNAs (CYP1B1-AS1, C9orf106) in tumors has not been reported. In our previous study, we have established an AML related circRNA-lncRNA-miRNA-mRNA ceRNA network based on the differentially expressed RNAs and the target relationship among circRNA-miRNA, lncRNA-miRNA, and miRNA-mRNA. Through comparing the previous results and the new discovery in this study, we noticed that lncCYP1B1-AS1 were involved in both studies, indicating that lncCYP1B1-AS1 may perform an important function in regulating the intracellular biological process and extracellular microenvironment of AML cells, and it had great potential and research value as a powerful prognostic marker (46). Zhang at al. identified 10 RNAs (LINC00471, hsa-mir-100, hsa-mir-150, ANP32E, ERMP1, MYO1B, PAPD7, PTGIS, TERF1, and VEGFA) to be ceRNAs closely related to childhood AML. Even prognostic RNAs in Zhang’s study are different from those in our study. We speculate that it may be due to the childhood AML remarkably differing from adult AML in karyotype, therapeutic strategy, and therapeutic effects. Still and all, it reminds us to consider more general markers for both types of AML and excavate more accurate markers for various subtypes (47).The abovementioned confirms that the ceRNA network established in this study plays an important role in the remodeling of the AML microenvironment, and has high prognostic value.

Chimeric antigen receptor immune cell therapy brings new hope for the treatment of leukemia and myeloma, and confirms that the tumor treatment using normal immune cells to penetrate and change the abnormal TME immune cell populations is effective and feasible. To systematically reveal the immune microenvironment of AML, we also used CIBERSORT algorithm to identify TME infiltrating immune cells, verify their prognostic value, and explore the relationship between TME immune cells and hub prognosis ceRNA. The results showed that the higher the proportion of M2 macrophages infiltration was, the poorer the prognosis (P = 0.002), and the lower the proportion of naive B cells (P <0.001) and helper follicular T cells (P = 0.016) would be, resulting in the lower the survival rate of the patients. In addition, the increased proportion of γδT cells, activated CD4 memory T cells and memory B cells also predicted the poor outcome of AML. Macrophages can be divided into M1 type that suppresses tumors and M2 type that promotes tumors. TME can induce the differentiation of monocytes and mesenchymal stem cells into M2 macrophages and promote the malignant process of tumor. M2 macrophages can not only suppress the anti-tumor immune response, but also promote tumor proliferation, invasion, migration and angiogenesis (48). Through analyzing a variety of blood tumors by CIBERSORT algorithm, Xu et al. found that the proportion of M2 macrophages in the AML microenvironment was much higher than that of the normal control and even other tumors, and it also predicted the reduced survival rate of patients and the rapid recurrence (49). In another study which identifies infiltrating lymphocytes in TME by CIBERSORT algorithm, γδT cells were found significantly increased in various hematological tumors, such as M3-AML, chronic myeloid leukemia, B-cell acute lymphoblastic leukemia, and could promote tumor progression by promoting tumor-related inflammatory response and mediating the formation of immunosuppressive TME (50). It is consistent with the results that proportions of TME infiltrating of M2 macrophages and γδT cells determines the outcome of AML in our study. Besides, we found that EPB41L3, COL2A1, CYP1B1-AS1, and C9orf106 are all highly correlated with immune infiltration, which is consistent with various studies. For example, Wendell et al. found that COL2A1 was bound up with cytotoxic lymphocyte immune signature and T-cell trafficking (51). It coincides with the finding of this study that there is a positive correlation between COL2A1 and naive CD4 T cells, confirming that COL2A1 is involved in the regulation of AML immune microenvironment.

Nevertheless, there are still some inescapable deficiencies in this study. On the one hand, due to the limitation of the existing data amount, the comparative study of different FAB classification and cytogenetic risk groups could not be completed, suggesting that more expression data is urgently needed for more accurate analysis. On the other hand, although we correlated ceRNA network with AML microenvironment through layers of verification, the role of multiple hub ceRNAs in AML is still not completely clear, and the exploration and verification of the functions of these RNAs is just beginning.

## Conclusion

In summary, our research combined ESTIMATE and CIBERSORT algorithms to identify AML TME related genes and immune cells, screened out ceRNA network that have high prognostic predictive power and highly TME association through multiple rigorous survival models and survival analysis. This study proposed a new way to reveal the role of TME in AML from the perspective of post transcriptional regulation, and contributed to exploring more diverse and effective AML biological markers.

## Data Availability Statement

The datasets analyzed for this study can be found in The Cancer Genome Atlas database (https://portal.gdc.cancer.gov/) and GEO (GSE142699–GPL26945, GSE71014–GPL10558) (https://www.ncbi.nlm.nih.gov/geo).

## Author Contributions

Conceptualization, YC and XW; methodology, PQ, CXL, SW, and LJ; software, XW, HD, QW and XS; validation, XW, YRL, and LY; formal analysis, YC, PQ and YS; investigation, YL, CYL, and CL; resources, XW and ZW; writing—original draft preparation, YC, CXL, and XW; writing—review and editing, XW and ZW. All authors have read and agreed to the published version of the manuscript.

## Funding

This work was supported by The National Key R&D program of China (2018YFC1106000).

## Conflict of Interest

The authors declare that the research was conducted in the absence of any commercial or financial relationships that could be construed as a potential conflict of interest.
