# Supplementary material for: Tumor Microenvironmental Competitive Endogenous RNA Network and Immune Cells Act as Robust Prognostic Predictor of Acute Myeloid Leukemia
Source: Front Oncol. 2021 Apr 9;11:584884. doi: 10.3389/fonc.2021.584884 (PMC8063692; doi:10.3389/fonc.2021.584884)
Supplement: Supplementary file 1 [file DataSheet_1.docx]

**Supplementary Figures:**


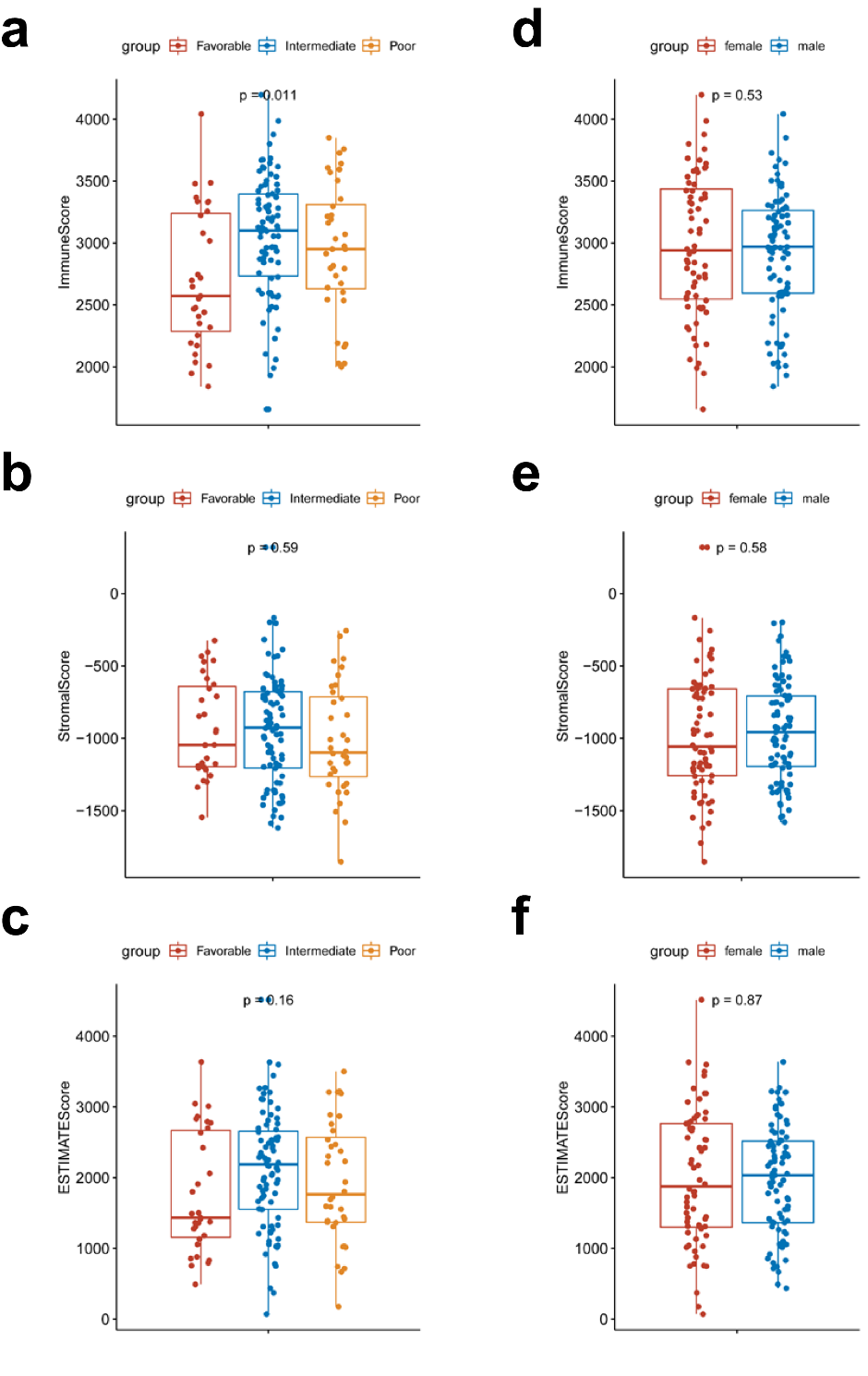


**Supplementary Figure 1. Identifying the relationship between TME scores and cytogenetic risk classifications and gender of patients. (a-c)** Distribution of immune, stromal and ESTIMATE scores in different cytogenetic risk classifications: the immune scores were significantly different among different cytogenetic risk groups (P =0.011), and there was no significant difference in stromal scores and ESTIMATE scores (P>0.05); **(d-e)** There was no significant differences between three scores and patients gender (all P values>0.05).

**
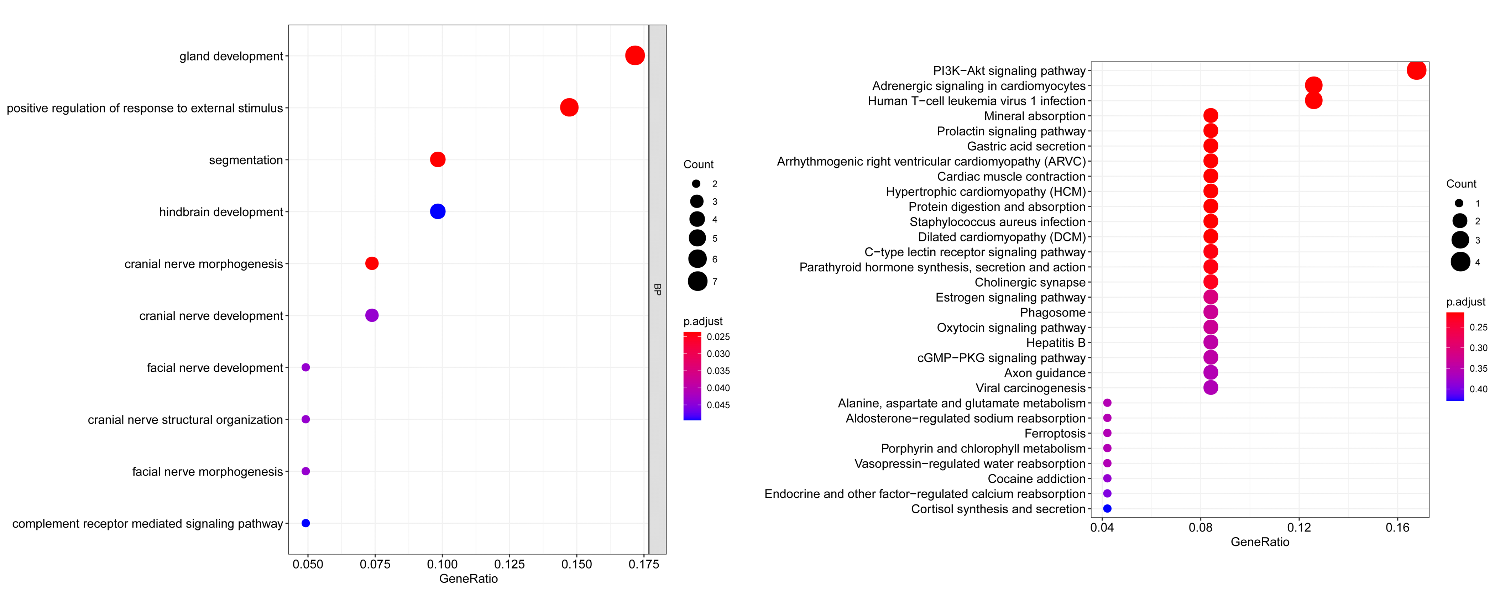
**

**Supplementary Figure 2.GO and KEGG enrichment analysis annotating the function of 43 preliminary identified hub mRNAs.**


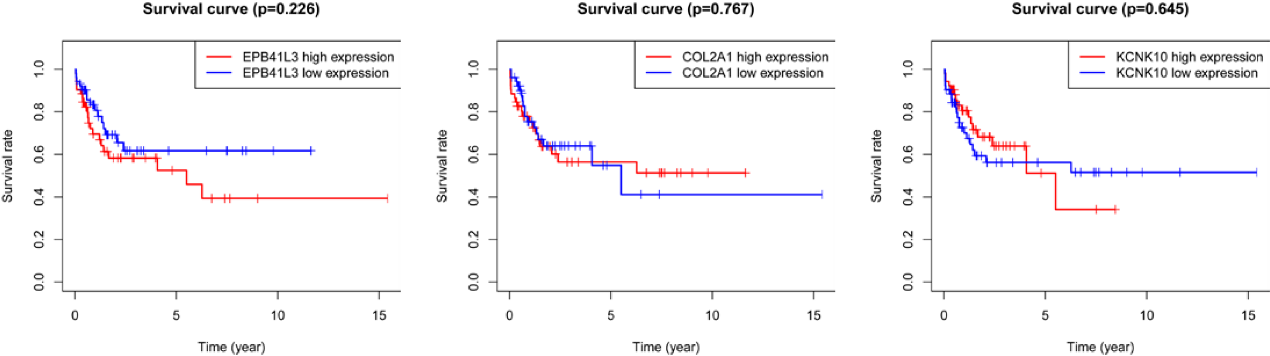


**Supplementary Figure 3. The validation of KCNK10, EPB41L3, and COL2A1 survival analysis by** **GSE71014 microarray.** The validation results were basically consistent with the TCGA database. The survival rate of AML patients with high EPB41L3 expression was significantly reduced, and survival rate AML patients with low COL2A1 expression was significantly reduced. There was no significant difference in survival rate among AML patients with different KCNK10 expression levels, while the P values were all greater than 0.05.


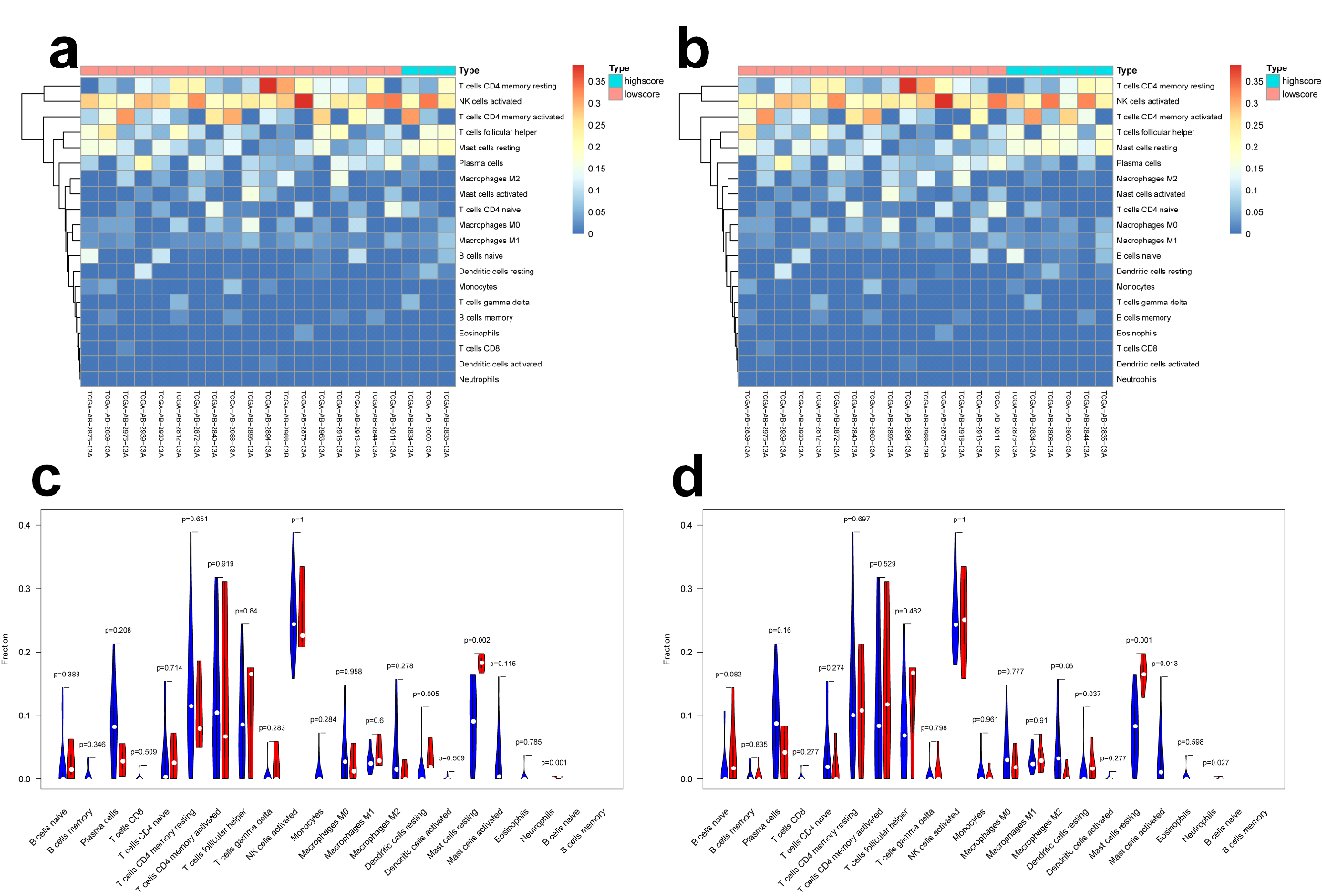


**Supplementary Figure 4. Heatmap and violin plot of immune cells within different immune and stromal score groups. (a)** Heatmap of the immune cells infiltration between the high and low **immune** group samples; **(b)** Heatmap of the immune cells infiltration between the high and low **stromal** group samples; **(c)** Violin plot of the immune cells infiltration between the high and low **immune** group samples: resting dendritic cells (P=0.005), resting mast cells (P=0.002) and neutrophils (P=0.001) showed significant differences between different immune group samples; **(d)** Violin plot of the immune cells infiltration between the high and low **stromal** group samples: resting dendritic cells (P=0.037), resting mast cells (P=0.001), activated mast cells (P=0.013) and neutrophils (P=0.027) are significantly different between samples in different matrix groups.


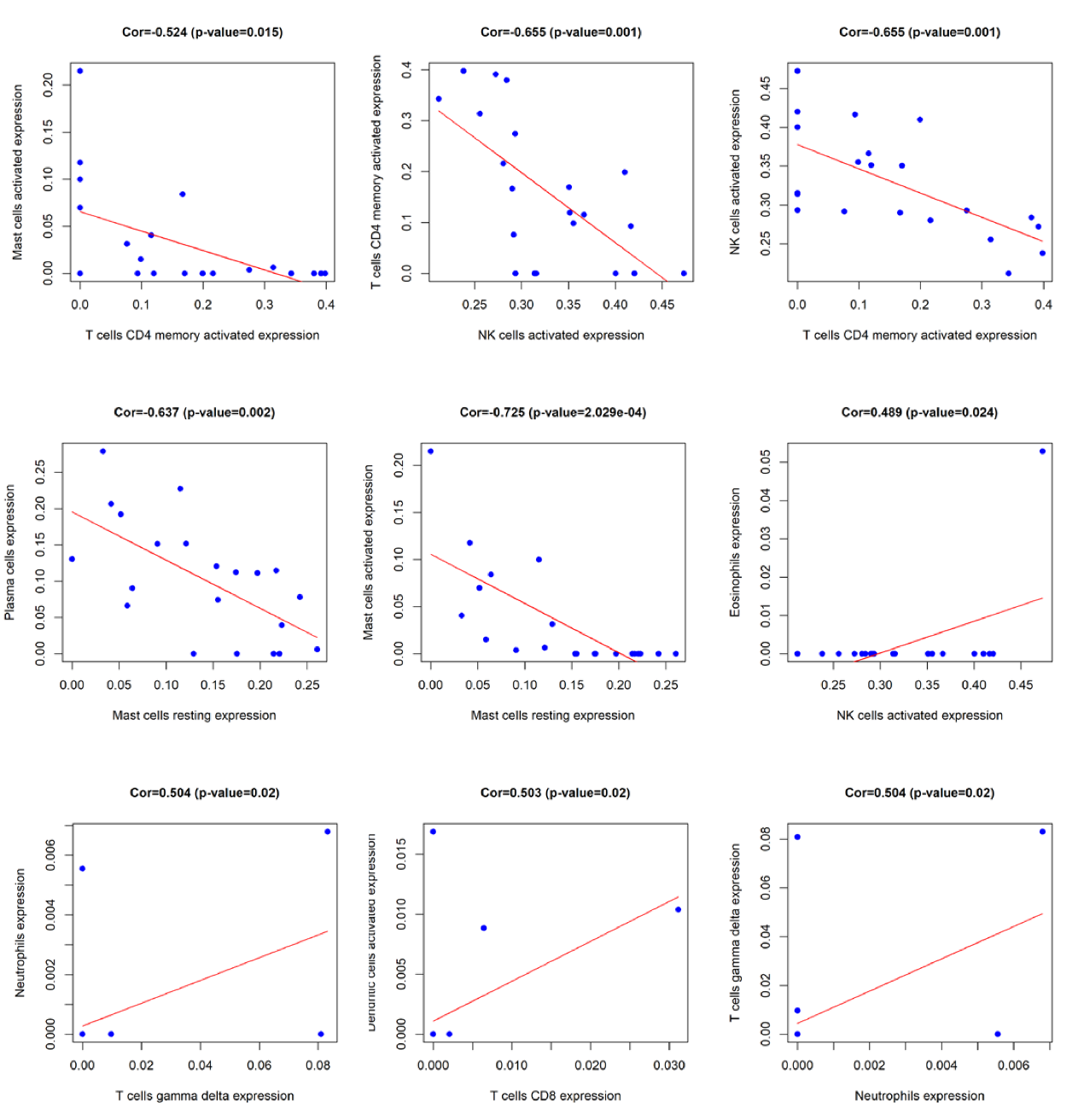


**Supplementary Figure 5. Identifying the correlation between TME infiltrating immune cell.** Activated memory T cells, activated mast cells, activated NK cells, activated CD4 memory T cells, plasma cells, resting mast cells and other immune cells were significantly correlated with each other (P < 0.05).


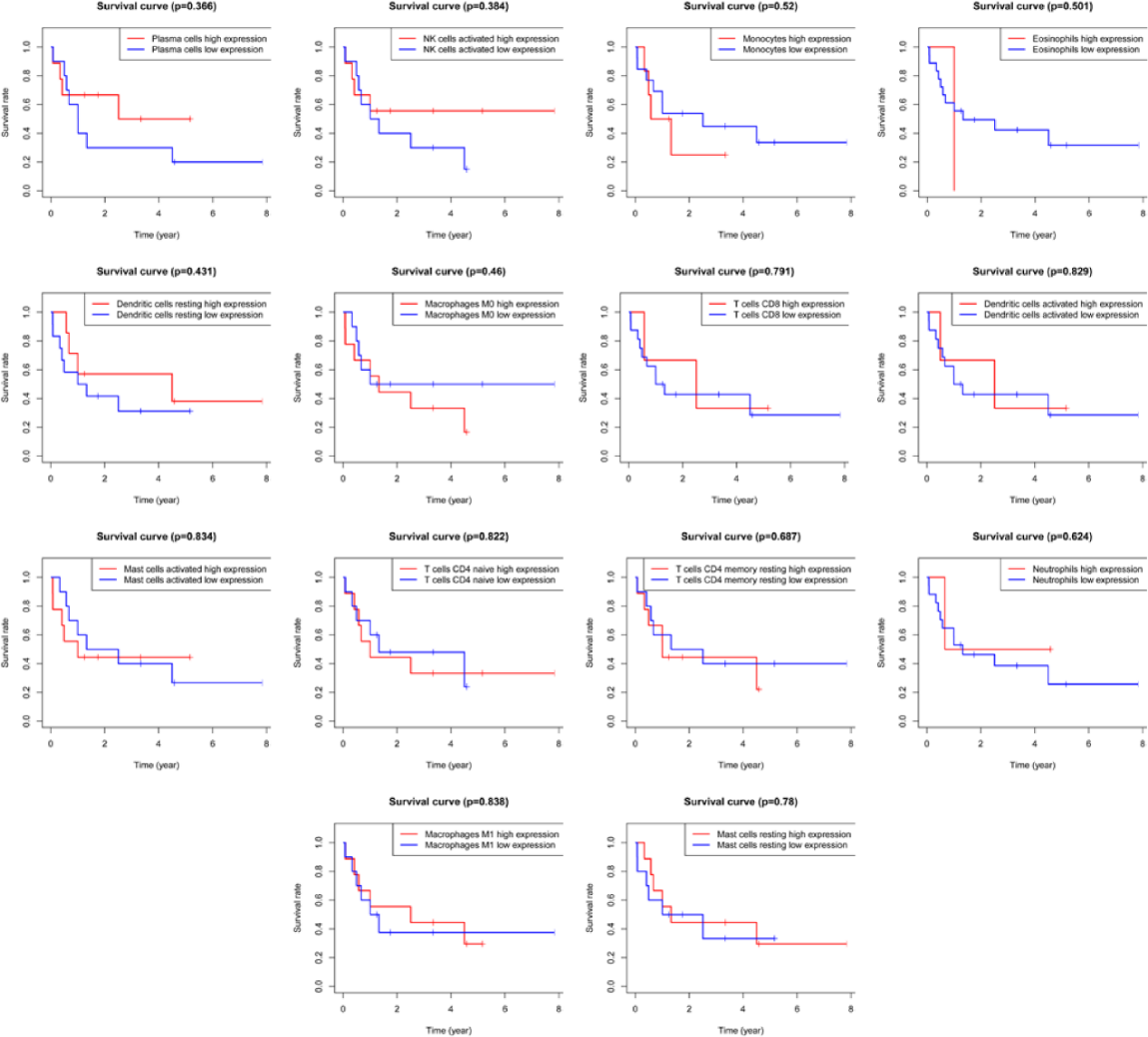


**Supplementary Figure 6. The relationship between TME infiltrating immune cells and prognosis was identified based on TCGA database.** Plasma cells, activated NK cells, monocytes, eosinophils, resting dendritic cells and other immune cells can all reflect the prognosis of patients to a certain extent (P values>0.05).


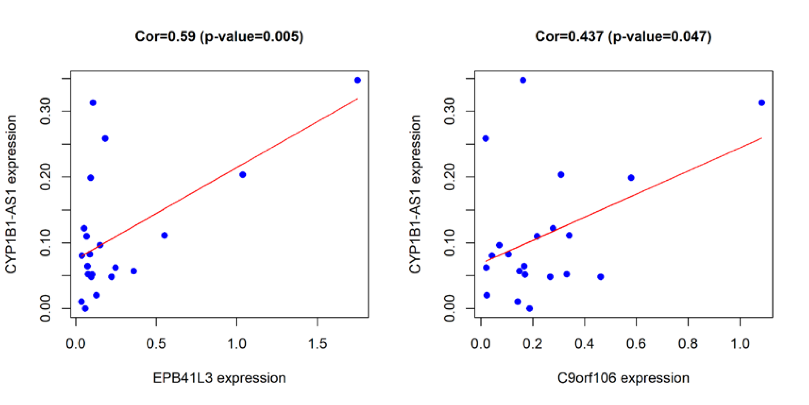


**Supplementary Figure 7. Correlation between hub prognostic ceRNA network components:** CYP1B1-AS1 and EPB41L3 (P=0.005), CYP1B1-AS1 and C9orf106 (P=0.047) were significantly positively correlated.
